# Ultra-lightweight superconducting wire based on Mg, B, Ti and Al

**DOI:** 10.1038/s41598-018-29354-1

**Published:** 2018-07-25

**Authors:** P. Kováč, I. Hušek, A. Rosová, M. Kulich, J. Kováč, T. Melišek, L. Kopera, M. Balog, P. Krížik

**Affiliations:** 1 Institute of Electrical Engineering, Slovak Academy of Sciences, Dúbravská cesta 9, 84104 Bratislava, Slovakia; 2 Institute of Materials and Machine Mechanics, Slovak Academy of Sciences, Dúbravská cesta 9, 84513 Bratislava, Slovakia

## Abstract

Actually, MgB_2_ is the lightest superconducting compound. Its connection with lightweight metals like Ti (as barrier) and Al (as outer sheath) would result in a superconducting wire with the minimal mass. However, pure Al is mechanically soft metal to be used in drawn or rolled composite wires, especially if applied for the outer sheath, where it cannot provide the required densification of the boron powder inside. This study reports on a lightweight MgB_2_ wire sheathed with aluminum stabilized by nano-sized γ-Al_2_O_3_ particles (named HITEMAL) and protected against the reaction with magnesium by Ti diffusion barrier. Electrical and mechanical properties of single-core MgB_2_/Ti/HITEMAL wire made by internal magnesium diffusion (IMD) into boron were studied at low temperatures. It was found that the ultra-lightweight MgB_2_ wire exhibited high critical current densities and also tolerances to mechanical stress. This predetermines the potential use of such lightweight superconducting wires for aviation and space applications, and for powerful offshore wind generators, where reducing the mass of the system is required.

## Introduction

Although many superconducting elements and compounds have been discovered^1^, only few of them can be used for thermally and mechanically stabilized long length wires with high current densities. Instead of high power cables and high field magnets, superconducting wires make possible the design of powerful and lightweight superconducting stators and rotors for aircraft engines and generators^2,3^. Lightweight superconducting wires are also attractive in other specific areas, where the total mass is extremely important, e.g. powerful wind turbines^4–6^ or any space applications^7,8^. Since the discovery of superconductivity in the lightest superconductive compound MgB_2_^9^, extensive efforts have been expended in the development of practical composite wires made mostly by powder-in-tube (PIT) processes and in the enhancement of their superconductive properties, particularly the critical current density (*J*_c_) and the upper critical field (*B*_c_). Low cost MgB_2_ superconductor wires operated at 4–25 K can lower the upfront and ongoing operational costs of superconducting systems. It was found that sheath materials play an important role in the determination of transport properties of the wires made by powder in tube (PIT) technique^10^. Cu is an ideal thermally stabilizing metal for low-*T*_c_ superconducting wires. In the case of MgB_2_ wires, the Cu reaction with MgB_2_ has to be inhibited due to a possible radical reduction of the transport current density. Therefore, a protective (i.e. diffusion) barrier has to be used (e.g. Fe, Nb or Ta) in order to avoid any reaction, namely the one between Cu and Mg^11^. Ti sheathed MgB_2_ wires were tested initially by Allessadrini^12^, and Ti barriers were then successfully applied for multicore MgB_2_ wires stabilized by Cu^13,14^. Al may also be an appropriate sheath material for MgB_2_ superconductor due to its high electrical and thermal conductivity, low cost, magnetism, and good formability. However, pure Al is mechanically soft metal to be used in drawn or rolled composite wires, especially if applied for the outer sheath, where it cannot provide the required densification of the boron powder. While Al alloys can offer improved mechanical properties, the conductivities and formability are markedly deteriorated. Furthermore, the solidus temperatures of Al alloys are much lower compared with the melting point of pure Al, which makes even more difficult the formation of MgB_2_ by the heat treatment of Mg and B components at ≈650 °C. The first experiment with MgB_2_/Al tape superconductor was made by an *ex-situ* PIT method without final heat treatment, but it does not allow reaching high critical current density^15^. Several other experiments to stabilize MgB_2_ wire with pure Al were also performed^16,17^, but the stabilization was not effective enough. Also, another solution with Al bonding on the Ti sheathed wire was not successful due to intensively oxidized surfaces of both Al and Ti^18^. Thermally stable ultrafine-grained Al stabilized by a small content of nano-scale Al_2_O_3_ formed *in situ* in Al matrix, named HITEMAL (high temperature aluminum), was produced by a powder metallurgy approach^19,20^. HITEMAL shows attractive mechanical and recently also electrical properties at low temperatures^21^. The first attempt to make an Al-stabilized MgB_2_ wire was made using Ta diffusion barrier and HITEMAL outer sheath^22^, which demonstrated the possible production of Al-sheathed MgB_2_ wires. It allowed to verify the utilization of Al + Al_2_O_3_ outer sheath for MgB_2_ wire and to show what superconducting properties, especially current densities, can be reached in medium magnetic fields. Ta barrier is really heavy material (16.69 g/cm^3^) for lightweight composite wire, but it has been used due to minimal reaction with Al + Al_2_O_3_ during the final heat treatment.

In this work we present original manufacturing method and properties of ultra-lightweight superconducting wire prepared by internal Mg diffusion process (IMD) into the B, which utilizes the lightest superconducting compound (MgB_2_ with 2.5 gcm^−3^) combined with the lightweight composite sheath (Al + 1.37 vol.% Al_2_O_3_ with a density of ~2.7 gcm^−3^) and light metallic barrier material (Ti with a density of 4.5 gcm^−3^). Due to lowered melting point of Al + 1.37 vol.% Al_2_O_3_ (∼ 652 °C)^22^ in comparison to pure Al (660 °C) and close to melting of Mg (650 °C), really specific heat treatment is needed for MgB_2_/Ti/HITEMAL wire. It should allow: (i) the fast formation of dense MgB_2_ phase^23^, (ii) limited Ti/Al interaction and (iii) keeping the mechanical strength of Al + Al_2_O_3_ sheath.

## Results

**Figure 1:**
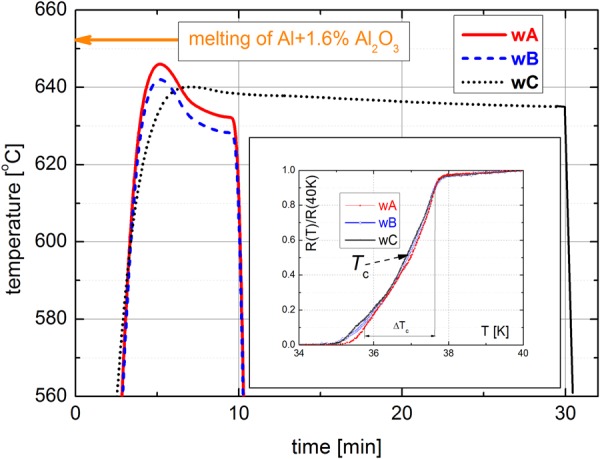
The pattern of final heat-treatments applied for wires wA, wB and wC. The insert shows the resistive transitions of compared wires.

**Figure 2:**
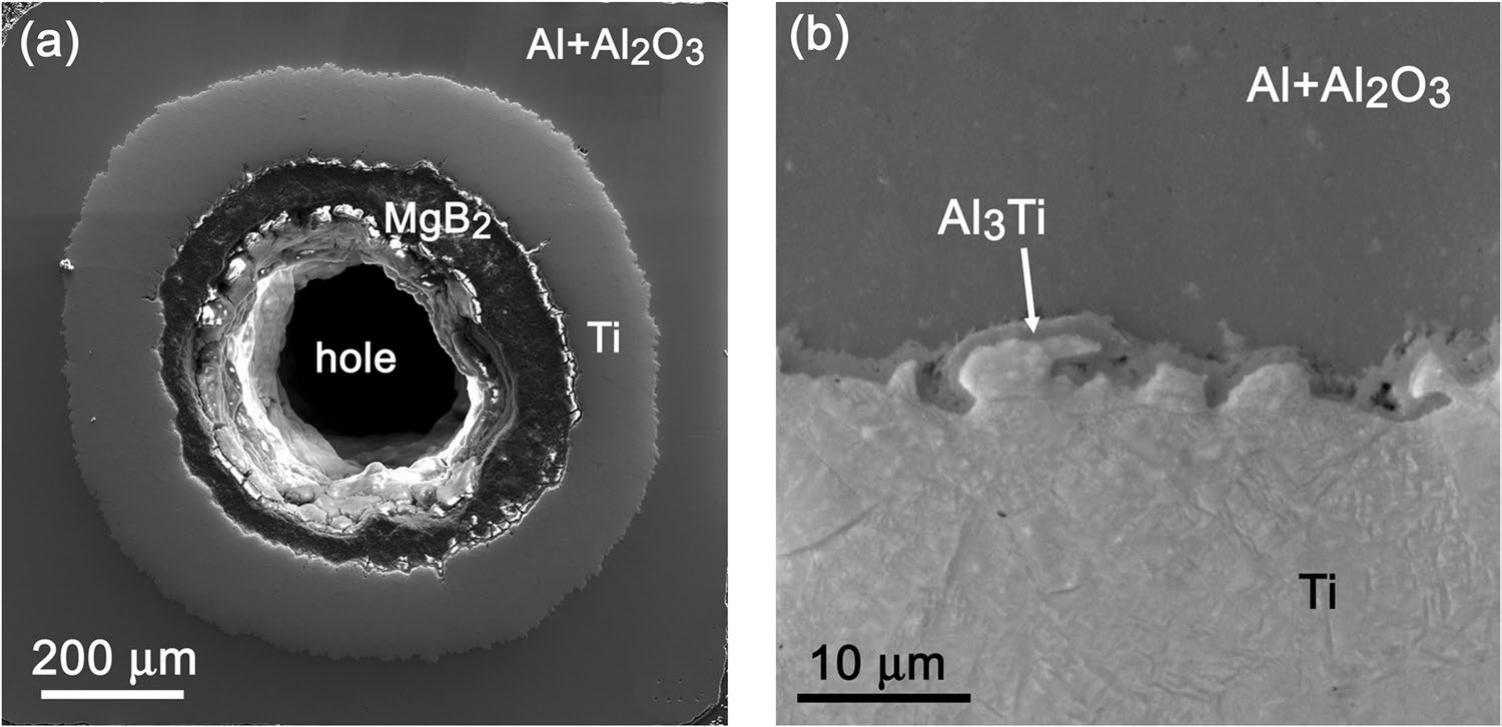
Transversal cross-section image of a heat-treated wB wire (**a**) and a detail of an Al_3_Ti reaction layer at the Ti/Al interface of the wB wire (**b**).

It was shown that the melting point of Al + 1.37 vol.% Al_2_O_3_ sheath is relatively low ~652 °C^22^ while the temperature close to 650 °C is required for the fast formation of a dense MgB_2_ phase^23^. This could cause undesirable changes (e.g., melting or recrystallization) of the Al_2_O_3_ stabilized Al sheath. Therefore, fast ramp heat treatments (~25 °C/min) with the setting temperature 628–635 °C and overshoot up to 640–646.5 °C were applied for as-deformed Mg/B/Ti/HITEMAL wires named as wA, wB and wC, see Table 1 and Fig. 1. A transversal cross-section image of the wB wire is shown in Fig. 2(a), where the central hole (at the place of the original Mg core) and formed MgB_2_ layer of thickness ∼100 µm are well visible. It correlates with the kinetic of MgB_2_ layer formation presented by Li at al. who have calculated the time needed for the for Mg + B reaction^24^. Our previous experiments have confirmed this model and showed the optimal time of 8 minutes for HT at 635 °C and overshoot 654 °C^23^. Figure 2(b) shows a thin intermetallic reaction layer with a thickness of ∼1 µm at the Ti/Al interface of the wB wire subjected to heat treatment (HT) temperature 628 °C/10 min.

**Table 1:** Heat treatment conditions with initial short temperature overshoot (*T*_max_) of wires wA-wC and the corresponding Al_3_Ti interface layer thickness, the HITEMAL sheath micro-hardness (HV_0.005_), the irreversible strain (*ε*_irr_) and stress (*σ*_irr_).

Wire	HT [°C/min]	*T*_max_ [°C]	Al_3_Ti layer [µm]	HV_0.005_ [GPa]	*ε*_irr_ [%]	*σ*_irr_ [MPa]
wA	632/10	646.5	∼1	∼43	0.166	141
wB	628/10	642.5	∼1	∼56	0.210	172
wC	635/30	640.0	∼4	∼68	0.342	214

The local EDS analysis confirmed Al_3_Ti phase, which is in good agreement with other studies^25^. A comparable Al_3_Ti layer of a similar thickness was observed for the wA and wB wires that were heat treated for 10 min, while for the wC wire that was annealed for 30 min, the layer increased to ∼4 µm (see Table 1). Formation of the Al_3_Ti phase at the Ti/Al interface may significantly decrease thermal and electrical transport between the MgB_2_ core and outer Al + Al_2_O_3_ sheath. To minimize the Al_3_Ti phase formation, a short heat treatment regime with a very fast initial ramp is preferred.

The critical current densities of the compared wires wA-wC were determined from the magnetic loops by using Bean’s critical state model to establish a relationship between the width of the hysteresis loop Δ*m* and the critical current density. Assuming a full penetration of the measured sample by a magnetic field, the particular form of the formulae relating Δ*m* to *J*_c_ can be derived with regard to the current flow geometry. In the case of a cylindrical MgB_2_ core, the critical current density is obtained according to:

$$\begin{array}{c}{J}_{c}=\frac{3}{d}{\rm{\Delta }}M\,{\rm{for}}B\,\mathrm{applied}\,{\rm{parallel}}\,{\rm{to}}\,{\rm{the}}\,{\rm{wire}}\,{\rm{axis}}\,(B||)\,\,{\rm{and}}\\ \,{J}_{c}=\frac{3\pi }{4d}{\rm{\Delta }}M\,{\rm{for}}\,B\,{\rm{applied}}\,{\rm{perpendicular}}\,\mathrm{to}\,\mathrm{the}\,{\rm{wire}}\,{\rm{axis}}\,(B\,\perp \,)\end{array}$$

where Δ*M* is the width of the hysteresis loop divided by the volume of the MgB_2_ core, and *d* is the core diameter. For the wires made by the IMD process resulting in an annular MgB_2_ core shown in Fig. 1(a), all formulae must be multiplied by a factor considering the hollow core geometry^26^. The factor is $(1-\frac{{d}_{I}^{2}}{{d}_{O}^{2}})/(1-\frac{{d}_{I}^{3}}{{d}_{O}^{3}})$, where *d*_*I*_ and *d*_*o*_ is the inner and outer diameter of the MgB_2_ core and the formula for *J*_c_ is:

$${J}_{c}=\frac{3{\rm{\Delta }}M}{{d}_{O}}\frac{1-\frac{{d}_{I}^{2}}{{d}_{O}^{2}}}{1-\frac{{d}_{I}^{3}}{{d}_{O}^{3}}}$$

**Figure 3:**
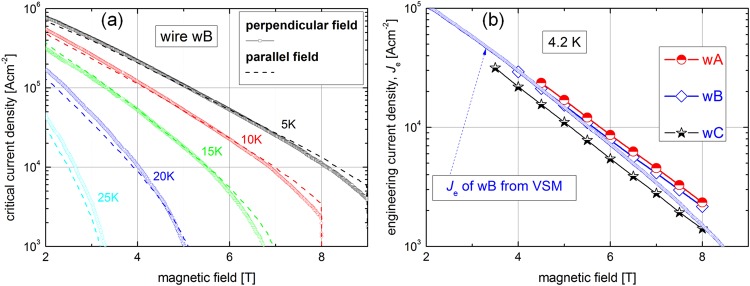
Critical current densities of the wB wire obtained from the VSM measurement (**a**) and *J*_e_(*B*) from the transport DC measurement of wires wA, wB and wC at 4.2 K (**b**) including *J*_e_ of wB wire from VSM measurement.

Filament dimensions *d*_*I*_ and *d*_*o*_ in wB wire are 0.00423 and 0.00625 cm, respectively. Figure 3(a) shows the critical current densities of the wB wire measured by a vibrating sample magnetometer at external magnetic fields 1–9 T (in *B*|| and *B*⊥) and temperatures 5–25 K. *J*_c_(*B*, *T*) values of the wire in the perpendicular field are identical with *J*_c_ of a single core MgB_2_/Ti/Cu wire made by the IMD process and annealed at 640 °C/60 min^27^, which is a result of a sufficiently dense boron powder deformed inside the Al + Al_2_O_3_ sheath. The dotted lines present *J*_c_ values at a parallel field, which are very similar to the perpendicular one for temperatures 5–15 K, and only slightly lowered at temperatures above 20 K. A small *J*_c_ difference between the perpendicular field direction (with currents flowing along the tubular core) and parallel one reflects an excellent homogeneity of the MgB_2_ compound created by the IMD process. An opposite behavior with large *J*_c_ differences between *B*|| and *B*⊥ was observed in MgB_2_ made by *in-situ* PIT process, which was attributed primarily to a texture caused by wire deformation and resulting to different ‘porosity’ or ‘connectivity’ in longitudinal and transversal direction^28^. Figure 3(b) shows the transport engineering current densities (calculated to the whole cross-section of the wire) at 4.2 K for wA, wB and wC samples. The highest *J*_e_ is measured for the wA due to the *T*_max_ = 646.5 °C, which is close to the melting point of Mg^22^. The mechanism of presented IMD process at temperatures below 650 °C considers fast Mg diffusion into boron powder and subsequent creation of MgB_2_ phase^23,24^. The resistive transitions of compared MgB_2_ layers are similar (see the insert in Fig. 1), but the systematic decrease of critical temperature (*T*_c_ = 37.00 K - wA, 36.89 K - wB and 36.87 K - wC) and small widening of *R*(*T*) transition (Δ*T*_c_ = 1.90 K - wA, 2.03 K - wB and 2.15 K - wC) can be observed. It reflects the composition and purity of created MgB_2_ phase and correlates with *J*_e_ values shown by Fig. 3(b).

Only slightly lowered peak temperature of 642.5 °C for the wB wire resulted in around 10% lower *J*_e_ compared with that of the wA wire, but *J*_e_ of wC wire is lowered by 37% at field 6 T in comparison to wA. Figure 3(b) shows also the relation between the transport and magnetic *J*_e_ (from VSM), for the wB wire, where a falling off of the magnetic *J*_e_ from the transport one was observed. It can be rationalized considering a different current flow combined with no fully identical connectivity along and across the core axis.

**Figure 4:**
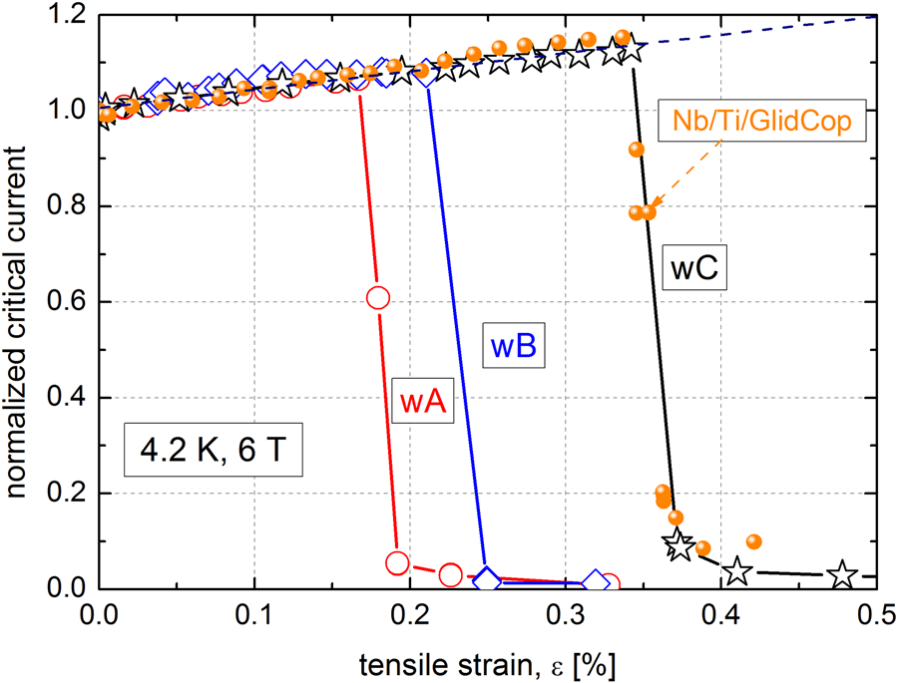
Strain tolerances of the wA, wB and wC wires to tensile stress at 4.2 K compared with a similar wire with a GlidCop outer sheath.

Changes in the critical currents of present wires subjected to axial tension at 4.2 K are shown in Fig. 4. Due to a larger thermal contraction of Ti and Al compared with that of MgB_2_, cooling down to 4.2 K results in compression stress which acts on the MgB_2_ layer and reduces the critical temperature and current^1,29^. Applied axial tension partially compensates the pressure stress and consequently critical current increases up to a level of irreversible strain (*ε*_irr_), where the breaking of brittle MgB_2_ leads to a radical degradation. The *ε*_irr_ value defines the maximum strain at which the current still remains reversible^29^. One can see a considerable effect of applied heat treatment on the irreversible strain in Figure 4. The wA wire with the highest peak temperature of 646.5 °C behaves mechanically as the weakest, and consequently the lowest *ε*_irr_ = 0.166% is measured due to the apparent softening of the outer sheath.

However, the wC wire annealed at the peak temperature of 640 °C has the highest *ε*_irr_ = 0.342%, which is even comparable with the strain limit of a single-core IMD wire reinforced with a GlidCop sheath, see the filled circles in Fig. 4. GlidCop is dispersion strengthened copper which was already effectively used for some MgB_2_ wires^23^. Table 1 shows the irreversible strain *ε*_irr_ and irreversible stress *σ*_irr_ measured for wA-wC, which are correlating well with the sheath microhardness HV_0.005_ – the lowest for wA ∼ 43 GPa and the highest for wC ∼ 68 GPa. It was already shown that strain and stress tolerances (*σ*_irr_ and *ε*_irr_) of MgB_2_ wires are dominantly affected by mechanical strength of the outer sheath^30^. Therefore, structural changes in outer sheath of wires wA-wC were examined by transmission electron microscopy.

**Figure 5:**
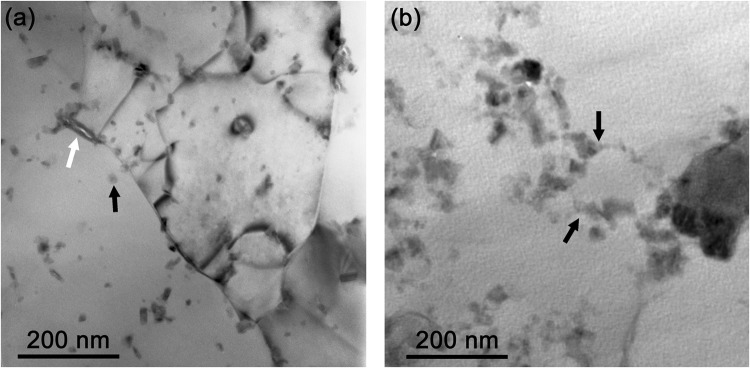
TEM bright field images of transversal-sections of the as-deformed Al + Al_2_O_3_ sheaths (**a**) and the heat-treated sheath of wA wire (**b**).

The as-deformed Al + Al_2_O_3_ consists of Al grains intensively elongated in the wire drawing direction and transversal structure shows a randomly distributed nanometric Al_2_O_3_ dispersoids, see Fig. 5(a). The nano-dispersoids stemmed from native amorphous (am)-Al_2_O_3_ layers on as-atomised Al powders^20^. The induced shear deformation broke up the Al_2_O_3_ layers into am-Al_2_O_3_ platelets during the cold working steps^21^, and some remnants of the fractured am-Al_2_O_3_ platelets remained at high angle grain boundaries, see the white arrow in Fig. 5(a). However, a majority of the am-Al_2_O_3_ platelets transformed into nanometric crystalline Al_2_O_3_ dispersoids during the cold working were found at both, the Al grain boundaries and within the Al grain interiors, see the black arrow in Fig. 5(a). During the final heat treatment Al grains coarsening is observed. High angle grain boundaries are preferentially eliminated with increasing temperature, but low angle grain boundaries are still stabilized by Al_2_O_3_ dispersoids and are sustained even higher annealing temperatures, see Fig. 5(b). The black arrows show co-localization of low angle grain boundaries with Al_2_O_3_ dispersoids in the wA wire sheath.

**Figure 6:**
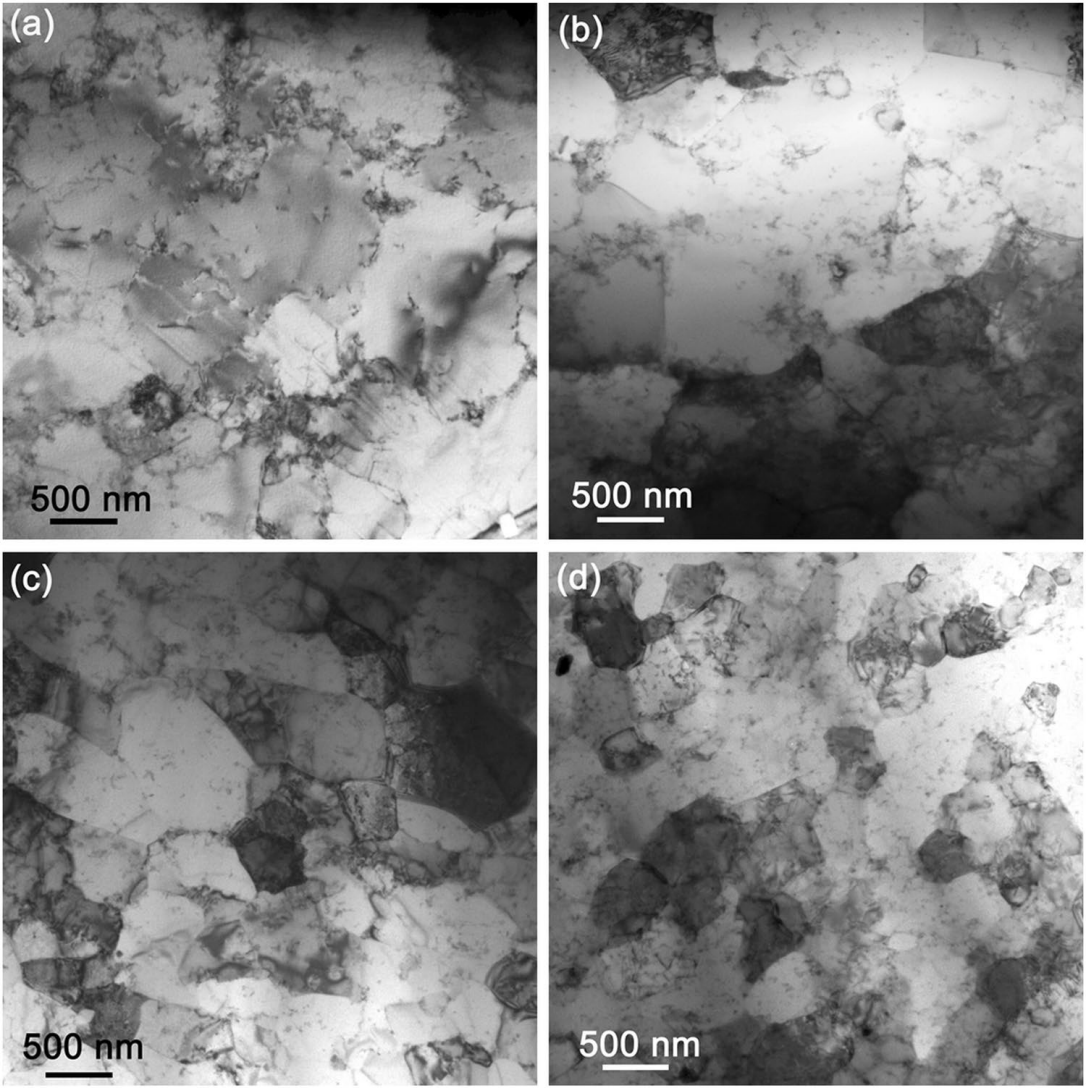
TEM bright field images of transversal-sections of the Al + Al_2_O_3_ sheaths of the wA (**a**), wB (**b**), wC (**c**) wires and of the as-deformed one (**d**).

Figure 6 shows TEM bright field images of transversal sections of the Al + Al_2_O_3_ sheath in the heat-treated wires wA - wC (a-c) in comparison to as-deformed one shown by Fig. 6(d). The TEM micrographs demonstrate the different microstructure upon annealing with the peak temperature between 640 °C and 646.5 °C. While Al grains of as-deformed wire have generally equiangular shape of averaged size *d*_av_ ∼ 470 nm, enlarged and/or elongated (not equiangular) grains are visible in wB and wC wires due to grains coarsening. High angle grain boundaries of Al + Al_2_O_3_ sheaths are yet well stabilized by Al_2_O_3_ dispersoids at heat treatment temperature *T*_max_ = 640 °C, see Fig. 6(c), where nearly doubled grain size *d*_av_ ∼ 950 nm in comparison to as-deformed sheath is found. The outer sheath of wC wire stays polycrystalline with the structure similar to the as-deformed one, see Fig. 6(d). The grain size structure of wB sheath shown by Fig. 6(b) is more affected by annealing only ∼10 °C below the melting of Al + Al_2_O_3_ and *d*_av_ ∼ 1380 nm was estimated for *T*_max_ = 642.5 °C. Figure 6(a) shows that grain boundaries in wA (5.5 °C bellow the melting of Al + Al_2_O_3_) are not more stabilized and a big Al grains with sub-grains and low angle grain boundaries with localized Al_2_O_3_ dispersoids are present. Consequently, the correct estimation of *d*_av_ for wA wire is not possible. Observed structural changes and grains coarsening leads to mechanical softening of heat treated Al + Al_2_O_3_ sheaths, which is accompanied by the decreased sheath micro-hardness (see Table 1) in comparison to not annealed Al + Al_2_O_3_ wire with HV_0.005_ ∼ 70 GPa^22^.

## Discussion

The presented microstructural study clearly illustrates that the different Al grain structure of the Al + Al_2_O_3_ sheaths strongly affects the wire responses to axial tension. The Al + Al_2_O_3_ is a suitable material for a sufficiently strong superconducting wire, but conditions of the final heat treatment have to be controlled very precisely. Figure 4 shows the different strain tolerances, which are strongly affected by the applied annealing influencing the sheath microstructure (see Figs 5 and 6). While the apparent critical current degradation in the wA wire occurred at the tensile stress of 141 MPa due to not more stabilized grain boundaries by Al_2_O_3_ dispersoids, the wC wire is able to withstand much higher stress of 214 MPa. Due to polycrystalline structure and grain size *d*_av_ ∼ 950 nm, the mechanical strength of the wC wire was by ~25% higher than determined for the wA wire, which is characterized by big grains with sub-grains and low angle grain boundaries. The averaged grain size of wB sheath (*T*_max_ = 642.5 °C) is ∼1380 nm, which is larger than for wC and consequently *σ*_irr_ = 172 MPa is measured, see Table 1. Similar correlation (sheath softening) is observed by the micro-hardness (HV_0.005_) data, which decreased from HV_0.005_ = 68 to 43 as the peak temperature increased from 640 to 646.5 °C, respectively (see Table 1). Nevertheless, the achieved HV_0.005_ = 43 for the Al-Al_2_O_3_ sheath of the wA wire remains still much higher than that of pure Al (HV_0.005_ = 27)^21^ due to a dense net of low angle grain boundaries effectively stabilized by Al_2_O_3_ dispersoids. The observed differences are attributed only to structural changes in the Al + Al_2_O_3_ material (and formation of thicker Al_3_Ti layer) subjected to different heat treatment. Therefore, precisely chosen heat treatment has to be applied to form a high current density MgB_2_ core along with a high strength Al + Al_2_O_3_ sheath and Ti diffusion barrier with a limited interfacial reaction at the sheath interface.

Calculation of conductor mass based on the MgB_2_/Ti/Al + Al_2_O_3_ can lead to at least 2.5 times weight reduction when compared with a typical MgB_2_/Nb/Cu wire of the same cross-sectional dimensions. This clearly outlines the potential of the lightest MgB_2_/Ti/Al + Al_2_O_3_ superconducting wire, when compared with any other metallic or ceramic superconductors. Consequently, presented MgB_2_ wire meets demanding requirements on electrical and mechanical properties of superconductors for efficient superconductive and light-weight applications.

## Methods

A single-core MgB_2_ wire was fabricated by internal magnesium diffusion (IMD) into a boron process. Pure Mg99.99% rod 2.9 mm in diameter was precisely positioned in the central axis of a Ti99.99% tube with 5.5 mm inner diameter and 7.2 mm outer diameter. The free volume between the Ti tube and Mg rod was filled by a B99.8% powder (<1 µm size) in a glove-box under pure Argon atmosphere. The Mg/B/Ti composite was rotary swaged down to 6.2 mm diameter, cleaned, and inserted into a HITEMAL tube with 6.3 mm inner diameter and 9.1 mm outer diameter, that was machined from an as-extruded Al + 1.37 vol.% Al_2_O_3_ rod^21^. The Mg/B/Ti/Al + Al_2_O_3_ composite rod was rotary swaged down to 7.5 mm and then groove rolled to a rectangular wire with a cross-section of 1.02 × 1.02 mm^2^. A heat treatment process was applied at 300 ^o^C for 30 min during the groove rolling process each time after reaching around 50% area reduction. The volume composition of the as-deformed wires corresponded to around 11% Mg, 12% B, 27% Ti, and 50% Al + Al_2_O_3_ outer sheath. The following final heat treatment was applied under Ar atmosphere at: (i) 632 °C for 10 min (wA wire); (ii) 628 °C for 10 min (wB wire); and (iii) 635 °C for 30 min (wC wire), with the peak temperatures of 646.5, 641.5 and 640 °C, respectively, see Fig. 1 and Table 1.

Hysteresis loops measured by a vibrating sample magnetometer (VSM) option in PPMS of Quantum Design system were recorded between −2 and +9 T with a constant field sweep of 6.3 mT/s in a temperature range of 5–25 K (at 5 K steps), and the field directed perpendicular and parallel to the wire axis. Using Bean’s critical state model, the critical current density *J*_c-mag_ was determined. Resistive transitions were measured by a standard four-probe method with DC current magnitude of 100 mA. Critical temperature (*T*_c_) and the width of transition (Δ*T*_c_) were determined from *R*(*T*) dependences shown by the insert in Fig. 1. Transport critical currents were measured at liquid He temperature and an external magnetic field from 4.0 to 8.0 T using standard DC measurement with 1 μVcm^−1^ criterion for *I*_c_ values. A free-standing short sample (∼50 mm) configuration was used for the tensile load tests of the wires at 4.2 K^29^. The electro-mechanical characteristics: *I*_c_ versus the tensile strain (*ε*) and the stress-strain curves *σ*(*ε*) were measured at a constant external magnetic field of 6 T. Scanning electron microscopy (SEM, JOEL 7600 F) with energy dispersive spectrometry (EDS, Oxford Instruments X-Max 50) was used to characterize polished transversal-sections of the heat-treated wires. Transmission electron microscopy (TEM) observations were made using JEOL JEM 1200FX microscope. TEM specimens were prepared by mechanical grinding and polishing followed by Ar beam ion milling using GATAN PIPS II. The transversal Al grain size (*d*_av_) was determined by image analyse of multiple bright filed TEM micrographs.
